# CONGENITAL AGENESIS OF THE INFERIOR VENA CAVA IN THE SETTING OF PRESENTING PULMONARY EMBOLISM: A CASE STUDY

**DOI:** 10.51894/001c.123039

**Published:** 2024-08-30

**Authors:** Victoria Brown

**Affiliations:** 1 Emergency Medicine Sparrow Main Hospital

## 61

### INTRODUCTION

Congenital absence of the IVC is a rare anomaly that is most often associated with deep vein thrombosis, but less likely to present with pulmonary embolism. Sometimes there is atresia of the retro hepatic segment of the IVC during embryogenesis. Typically, this anomaly is either found incidentally, or there is a thrombus, particularly if found in a younger person, that can indicate an underlying problem. This finding is usually best depicted on computed tomography with angiography (CTA) with venous phase imaging. Patients with a congenital absence of the IVC are at increased risk for thromboembolism due to reduced venous flow, venous hypertension and thrombophilia.

### CASE DESCRIPTION

The patient is 31-year-old male with past medical history of ADHD and schizophrenia presenting with new onset lower back pain & RLE swelling and pain with associated shortness of breath. He characterizes his lower back pain as “band-like” along the lower lumbar region. Physical exam notable for tenderness to palpation along back and right lower extremity edema. Initial vitals stable. Labwork unremarkable. Imaging revealed bilateral pulmonary emboli with RLE DVT. Patient was provided with IM 1 mg/kg dosage of Lovenox. Later transitioned to heparin gtt after 12 hours post Lovenox. Admission to hospitalist team for further evaluation and treatment of RLE DVT and bilateral pulmonary embolism. After admission - plan for vascular surgery or interventional radiology consultation for further recommendations.

### DISCUSSION

Agenesis of the IVC during embryogenesis leads to venous stasis, venous hypertension and thrombophilia that increases the risk for thromboembolism in the lower extremities. Thromboembolism of the lower extremities can then lead to an embolus travelling through collateral veins to the azygos vein, which drains into the superior vena cava to the heart and the lungs, causing a PE. Most commonly, a PE is a result of a thromboembolism originating from a DVT. In the case of congenital agenesis of the IVC, the thromboembolism travels via the azygos vein to the heart and to the lungs. Literature review revealed that PE is not a common presenting symptom, usually, the most common presenting symptom is a DVT in a younger patient.

